# Progressive Dissociation Between Reactogenicity and Immunogenicity After Four-Dose BNT162b2 Vaccination: A 36-Month Longitudinal Study

**DOI:** 10.3390/vaccines14040305

**Published:** 2026-03-28

**Authors:** Sanja Zember, Kristian Bodulić, Nataša Cetinić Balent, Alemka Markotić, Oktavija Đaković Rode

**Affiliations:** 1 Health Care Quality Unit, University Hospital for Infectious Diseases “Dr. Fran Mihaljević”, Mirogojska 8, 10000 Zagreb, Croatia; szember@bfm.hr; 2 Research Department, University Hospital for Infectious Diseases “Dr. Fran Mihaljević”, Mirogojska 8, 10000 Zagreb, Croatia; kbodulic@bfm.hr (K.B.); amarkotic@bfm.hr (A.M.); 3 Department of Virology, University Hospital for Infectious Diseases “Dr. Fran Mihaljević”, Mirogojska 8, 10000 Zagreb, Croatia; ncetinic@bfm.hr; 4 Faculty of Medicine, Catholic University of Croatia, 10000 Zagreb, Croatia; 5 Faculty of Medicine, University of Rijeka, 51000 Rijeka, Croatia; 6 School of Dental Medicine, University of Zagreb, Gundulićeva 4, 10000 Zagreb, Croatia

**Keywords:** BNT162b2 vaccine, COVID-19, immunogenicity, reactogenicity, booster vaccination, longitudinal study, healthcare workers

## Abstract

**Background/Objectives:** Understanding the relationship between reactogenicity and immunogenicity after repeated BNT162b2 vaccination is critical for optimizing vaccination strategies. This study quantified their progressive dissociation across four vaccine doses. **Methods:** We conducted a prospective longitudinal cohort study among Croatian healthcare workers vaccinated with BNT162b2 from January 2021 to January 2024. Anti-SARS-CoV-2 IgG antibodies were measured at 16 timepoints using chemiluminescent immunoassay. Local (pain, erythema, swelling) and systemic (fever, fatigue, headache, myalgia, arthralgia, nausea) reactions were recorded for 7 days using FDA toxicity scale. Correlations were analyzed with Spearman’s method and Bonferroni correction. Fourth-dose responses were predicted by exponential modeling. **Results:** Of 631 participants, 524 completed primary immunization, 418 received a third dose (173 with complete data), and 56 received a fourth dose (22 with complete paired data). Local reactions declined from 82.4% after the first dose to 42.9% after the fourth (*p* < 0.001). Systemic reactions peaked at 44.8% after the second dose, then decreased to 26.0% after the third and 19.6% after the fourth. In contrast, median antibody levels rose from 9910 AU/mL after the primary series to 29,002 AU/mL after the third and 38,274 AU/mL after the fourth. Correlations between reactions and antibody titer progressively weakened: *r* = 0.37 (95% CI 0.29–0.44, *p* < 0.001) after the primary series, *r* = 0.08 (95% CI −0.07 to 0.23, *p* = 0.30) after the third, and *r* = 0.04 (95% CI −0.39 to 0.45, *p* = 0.86) after the fourth dose. **Conclusions:** Progressive dissociation between reactogenicity and immunogenicity was observed across four BNT162b2 doses. Booster doses maintain robust antibody responses despite reduced reactogenicity, reinforcing that minimal side effects are consistent with sustained humoral responses.

## 1. Introduction

With more than 13.6 billion doses administered worldwide, COVID-19 vaccination represents the largest public health intervention in modern history [1]. Among the available vaccines, BNT162b2 (Pfizer-BioNTech) utilizes lipid nanoparticles to deliver N1-methylpseudouridine-modified mRNA encoding the SARS-CoV-2 spike protein [2]. Phase III trials reported 95% efficacy against symptomatic infection [3], findings later confirmed in real-world studies across Israel [4], the United Kingdom [5], and the United States [6].

Despite this strong initial protection, waning immunity soon emerged as a consistent phenomenon. Following the two-dose primary series, antibody levels declined by 82–93% within six months [7,8,9], both in healthcare workers [10] and in population-based studies [11]. This rapid decline led to widespread implementation of booster doses, which restored antibody titers to levels roughly three times higher than those achieved after the second dose [12]. While subsequent boosters also showed declining antibody levels, the kinetics appeared slower compared to the primary series [13,14]. Importantly, 2024–2025 data confirmed that boosters retained significant protection against severe outcomes, with vaccine effectiveness of 33% against emergency department visits and 45–46% against hospitalization among older adults [15]. However, studies of XBB.1.5-adapted formulations documented marked reductions in long-term effectiveness [16], underscoring the need for continuous vaccine updates.

Reactogenicity, the constellation of inflammatory symptoms after vaccination, has traditionally been viewed as a correlate of immunogenicity in conventional vaccines [17]. However, the unique properties of BNT162b2, including its pseudouridine modification, may alter this relationship [18]. In the pivotal trial [3] and subsequent pharmacovigilance data [19], reactogenicity increased from first to second dose, but booster studies demonstrated progressively weaker associations. Notably, the third dose produced fewer adverse events compared with the second, while the fourth dose remained well tolerated and maintained robust immunogenicity [20]. These findings suggest a dissociation between reactogenicity and immunogenicity with repeated dosing.

Parallel immunological adaptations further support this notion. Although causality remains unproven, several studies reported IgG4 class switching, with spike-specific IgG4 increasing from <1% after primary immunization to ~20% after the third dose [21,22]. This may reflect a tolerance mechanism to repeated antigen exposure. Importantly, a Spanish cohort study in 2024 linked elevated IgG4 with a 1.8-fold increased risk of breakthrough infections [23], emphasizing that antibody quality, not just quantity, is clinically relevant.

Despite extensive vaccine surveillance, the longitudinal relationship between reactogenicity and immunogenicity remains incompletely understood. Early studies identified a moderate positive correlation (r = 0.366; *p* < 0.0001) between systemic adverse event scores and antibody titers after the second dose [24], whereas others found no consistent associations [25]. A 2025 Australian randomized trial comparing fourth-dose mRNA and protein vaccines demonstrated higher immunogenicity with mRNA platforms despite greater reactogenicity [26], but specific correlation analyses were not reported. Critically, no study has systematically quantified this relationship across four consecutive doses in a multi-year follow-up with adequate statistical power.

This knowledge gap carries immediate implications for vaccine policy and clinical practice. The 2025 FDA requirement for placebo-controlled trials in younger adults [27], together with the updated Advisory Committee on Immunization Practices (ACIP) recommendations for additional doses in adults ≥ 65 years [28], highlights the importance for high-quality data on long-term vaccine responses. In particular, understanding whether minimal reactogenicity after repeated boosters compromises immunogenicity could influence vaccine acceptance and inform personalized vaccination strategies.

Based on these considerations, we hypothesize that repeated BNT162b2 vaccination induces a progressive dissociation between reactogenicity and immunogenicity. The primary objective of this 36-month longitudinal study was to quantify this correlation across four vaccine doses in a well-characterized cohort of Croatian healthcare workers. Secondary objectives were: (1) to determine whether successive boosters extend antibody half-life, (2) to evaluate whether individuals with hybrid immunity demonstrate earlier dissociation, and (3) to characterize the mathematical pattern of correlation decay.

## 2. Materials and Methods

### 2.1. Study Design and Participants

We conducted a prospective longitudinal cohort study at the University Hospital for Infectious Diseases “Dr. Fran Mihaljević”, Zagreb, Croatia, from 5 January 2021 to 31 January 2024. The study adhered to the Declaration of Helsinki [29] and was reported in accordance with STROBE guidelines [30] (Supplementary Materials, Table S1).

Ethics approval was obtained through expedited procedures for COVID-19 research. Initial approval was granted on 5 February 2021 (protocol no. 01-228-1-2021), with subsequent amendments covering the third dose (19 November 2021; protocol no. 01-1913-1-2021) and the fourth dose (13 December 2022; protocol no. 01-2404-6-2022). Additional approval was obtained from the School of Dental Medicine, University of Zagreb (27 February 2024; protocol no. 05-PA-2-25/2024). All participants provided written informed consent.

Healthcare workers were enrolled during the national vaccination campaign. Eligible participants were adults (>18 years) who received the BNT162b2 vaccine and consented to serial serological testing. Individuals vaccinated with other formulations, those with a documented allergic reaction to vaccine components, pregnant women at the time of primary vaccination, and participants with incomplete data were excluded. Demographic data, medical history, and occupational exposure were collected at baseline. Participants who completed primary vaccination were subsequently offered booster doses in line with national guidelines. Follow-up assessments were conducted at sixteen predefined time points: three weeks after the first dose; one, three, six, and nine months after the second dose; one week after the third dose, followed by one, three, six, and twelve months thereafter; and one week after the fourth dose, followed by one, three, six, nine, and twelve months thereafter.

SARS-CoV-2 infections were diagnosed either by RT-PCR or documented seroconversion. Infections were categorized as pre-vaccination (occurring prior to the first dose) or breakthrough (occurring ≥ 14 days after the completion of each vaccination phase). Disease severity was classified using World Health Organization (WHO) criteria [31]. Participants who developed breakthrough infection during follow-up were excluded from subsequent immunological analyses to prevent confounding by natural infection.

### 2.2. Vaccination Protocol

The BNT162b2 vaccine (30 μg in 0.3 mL) was administered intramuscularly into the deltoid muscle according to the manufacturer’s instructions and national guidelines [32]. Primary immunization consisted of two doses given 21 days apart. The third dose (first booster) was administered approximately nine months after the second dose, and the fourth dose (second booster) approximately twelve months after the third dose, following updated recommendations.

### 2.3. Adverse Event Assessment

Adverse events were monitored using structured questionnaires that assessed both local and systemic reactions. Local reactions included pain, erythema, and swelling at the injection site, while systemic reactions included fever ≥ 38 °C, chills, headache, fatigue, myalgia, arthralgia, and nausea. Each symptom was graded according to the FDA toxicity scale [33], where 0 indicated absence, 1 mild symptoms without activity limitation, 2 moderate symptoms associated with some limitation, and 3 severe symptoms that prevented normal activities. All symptoms were weighted equally due to absence of validated hierarchical severity scoring in this context. A composite adverse event (AE) score, ranging theoretically from 0 to 30, was calculated by summing the intensity of all local and systemic reactions. Questionnaires were distributed in both paper and electronic form via a secure hospital email system, and anonymous reporting was encouraged to minimize underreporting.

### 2.4. Serological Testing

Venous blood samples (5 mL) were collected at all predefined time points across the 36-month follow-up. Anti-SARS-CoV-2 IgG antibodies against the spike protein receptor-binding domain (RBD) were quantified using a chemiluminescent immunoassay (SARS-CoV-2 IgG II Quant, Abbott Laboratories, Abbott Park, IL, USA) [34]. All assays were performed by trained laboratory staff according to the manufacturer’s specifications, with quality controls included in every run. The assay demonstrated strong correlation with neutralizing antibody titers (r = 0.89 for PRNT50) [35]. Results were reported in arbitrary units per milliliter (AU/mL), with a positivity threshold of ≥50 AU/mL. The analytical range was 21–40,000 AU/mL, and samples exceeding the upper limit were diluted according to the manufacturer’s instructions. Serum samples were processed within four hours of collection and stored at −20 °C until analysis.

### 2.5. Statistical Analysis

Categorical variables were summarized as counts and proportions, while continuous variables were expressed as mean ± standard deviation in the case of normal distribution or as median and interquartile range in the case of non-normal distribution. The Shapiro–Wilk test was applied to assess normality. Correlations between reactogenicity and antibody levels were examined using Spearman’s rank correlation coefficient, with 95% confidence intervals derived via Fisher’s z-transformation. Trends across vaccine doses were evaluated using the Cochran–Armitage test for categorical variables and the Jonckheere–Terpstra test for continuous variables. The pattern of correlation decay across doses was characterized by fitting an exponential model, r(d) = r_0_ × exp(−λ × (d−2)), using nonlinear least squares regression. Parameters r_0_ and λ were optimized simultaneously, and the unconstrained approach provided more robust parameter estimation for small datasets compared to constrained fitting, offering improved predictive accuracy for the fourth-dose response. Given the limited number of empirical points (*n* = 3), the exponential model should be interpreted as descriptive rather than confirmatory.

Pre-specified subgroup analyses stratified participants according to infection history, distinguishing those with hybrid immunity from infection-naïve individuals. Odds ratios with 95% confidence intervals were calculated for binary outcomes, and fold-changes were used to compare peak antibody levels between doses. Multiple comparisons were corrected using the Bonferroni method. Sample size was calculated a priori to detect a moderate correlation (r ≥ 0.30) between adverse events and antibody titers with 90% power at a significance level of α = 0.05, requiring a minimum of 112 participants per group. The final sample size provided sufficient statistical power for analyses after the first three vaccine doses (primary series: *n* = 524; third dose: *n* = 173). The fourth-dose subgroup (*n* = 22) was underpowered to detect moderate correlations.

Antibody half-life was estimated using nonlinear mixed-effects modeling with first-order elimination kinetics [36]. Missing data were evaluated with Little’s MCAR test [37]. Sensitivity analyses included exclusion of participants with breakthrough infections, restriction to complete cases, and application of alternative correlation methods [38] to assess robustness. All statistical tests were two-tailed with a significance threshold set at α = 0.05. Analyses were performed using R version 4.3.3 (R Foundation for Statistical Computing, Vienna, Austria) [39] with the tidyverse package suite [40].

### 2.6. Bias Minimization

Several measures were implemented to minimize bias. Consecutive enrollment during the national vaccination campaign reduced selection bias. Recall bias was addressed by sending daily reminders for seven days after vaccination. Information bias was mitigated through the use of standardized questionnaires administered by trained personnel.

## 3. Results

### 3.1. Study Population and Flow

**Figure 1:**
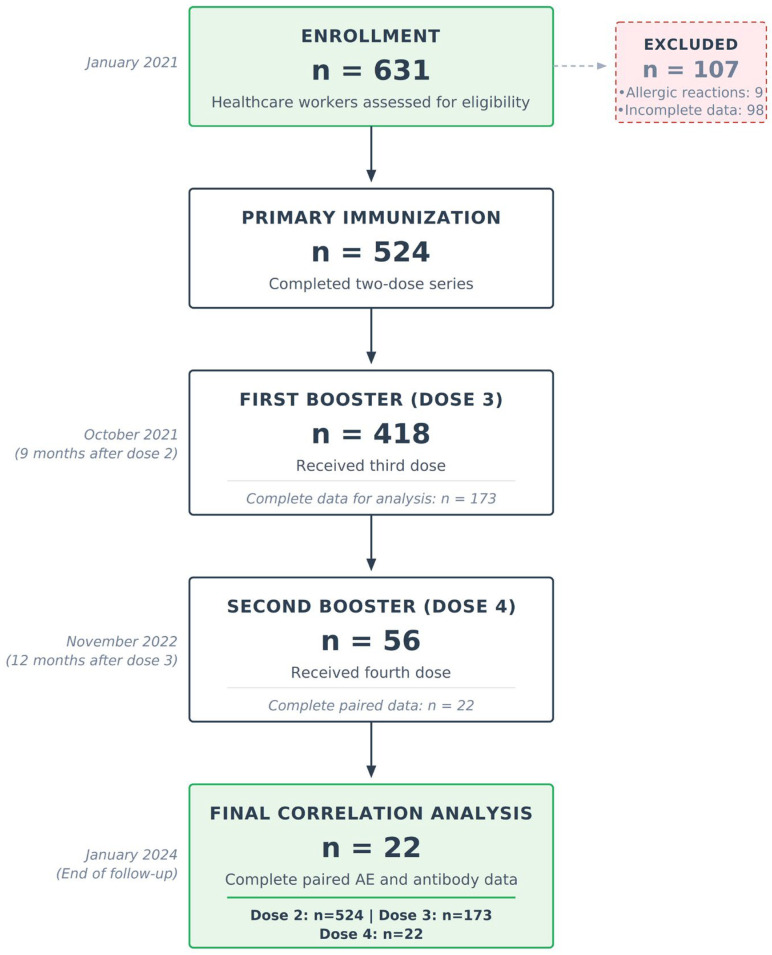
STROBE flow diagram of participant progression through the 36-month study (January 2021–January 2024).

Of the 631 healthcare workers initially enrolled, 524 (83.0%) completed primary vaccination and were included in the final analysis (Figure 1). A total of 107 participants were excluded: nine (1.43%) due to allergic reactions after the first dose and 98 (15.5%) owing to incomplete data. Allergic reactions consisted of generalized rash with pruritus in eight cases and transient neurological symptoms in one case. All participants received appropriate antiallergic therapy and recovered fully without lasting consequences. Baseline demographic and clinical characteristics are summarized in Table 1. The study cohort was predominantly female (81.7%), with a median age of 42 years (IQR 33–52 years).

**Table 2:** Progressive Dissociation between reactogenicity and immunogenicity across four BNT162b2 vaccine doses.

Parameter	Primary Series (n = 524)	Third Dose (n = 173)	Fourth Dose (n = 22) *
**Immunogenicity (median (IQR))**			
**Anti-SARS-CoV-2 IgG, AU/mL**	9910.5 (5234.2–18,645.3)	29,002.0 (17,951.1–45,440.5)	38,274.0 (33,216.5–62,452.5)
**Antibody half-life, days**	91 (87–95)	112 (104–121)	126 (117–136)
**Reactogenicity (mean (SD)**			
Composite AE score **	4.2 (2.8)	2.8 (2.1)	1.9 (1.6)
**Local score**	2.8 (1.9)	1.9 (1.5)	1.3 (1.2)
**Systemic score**	1.4 (1.3)	0.9 (1.0)	0.6 (0.7)
**Correlation (r) (95% CI)**			
**Observed correlation**	0.37 (0.29–0.44)	0.08 (−0.07 to 0.23)	0.04 (−0.39 to 0.45)
***p*-value**	<0.001	0.30	0.86
**Exponential model**			
**Model equation**	---	---	r(d) = 0.370 × e^−1.430 × (d−2)^
**Model fit (R^2^)**	---	---	0.9934
**Decline from first dose (%)**	---	78%	89%

* For fourth dose correlation analysis: Of 56 participants who received the fourth dose, 50 provided antibody measurements and all 56 reported adverse events. However, only 22 participants had complete paired data (both adverse events AND antibody levels at all required timepoints) necessary for correlation coefficient calculation. The antibody levels shown represent median values from the 50 participants with antibody data. ** Composite AE score = sum of all local and systemic symptoms (range 0–30). Notes: Composite AE scores are presented as mean (SD) based on approximately normal distributions confirmed by Shapiro–Wilk test, while antibody levels are presented as median (IQR) due to right-skewed distributions. Spearman correlation was used for all dose groups, given the ordinal nature of composite AE scores. CI calculated using Fisher z-transformation (R package DescTools). Participants who developed COVID-19 during follow-up were excluded from immunological analyses at timepoints after infection.

After completing the primary vaccination series, 418 participants (79.8%) proceeded to the third dose, among whom 173 provided complete paired datasets suitable for correlation analyses. Subsequently, 56 participants (10.7% of the original cohort) received the fourth dose. Within this subgroup, complete paired data for reactogenicity–immunogenicity correlation were available for 22 individuals; antibody titers were obtained from 50, while adverse events were reported by all 56. The pattern of missing data was assessed using Little’s MCAR test and confirmed to be missing completely at random (*p* = 0.423). This substantial reduction in sample size at the fourth dose limits precision of correlation estimates and may introduce selection bias.

Breakthrough infections occurred at multiple stages during follow-up. Between the second and third doses, 76 participants (14.5%) were infected; between the third and fourth doses, 115 participants (27.5% of third-dose recipients) experienced breakthrough infections; and following the fourth dose, three cases (5.4%) were reported. All infections were classified as mild or asymptomatic according to WHO severity criteria [31].

Of 631 healthcare workers assessed for eligibility, 524 (83.0%) completed primary immunization after excluding 107 participants due to allergic reactions (n = 9) and incomplete data (n = 98). During follow-up, 418 participants (79.8%) received the third dose in October 2021, of whom 173 retained complete adverse event and antibody data for analysis. The fourth dose in November 2022 was administered to 56 participants (10.7% of total cohort), with 22 participants providing complete paired data required for correlation analysis between reactogenicity and immunogenicity.

### 3.2. Reactogenicity Patterns

Local reactions demonstrated a clear progressive decline across successive doses, decreasing from 82.4% after the first dose to 42.9% after the fourth dose (*p* < 0.001 for trend; Table 1). Systemic reactions, however, followed a different trajectory: they peaked at 44.8% after the second dose and subsequently declined to 19.6% after the fourth dose. Severe adverse events (Grade 3) were most frequent following the second dose (12.4%) but were rare after the fourth (1.8%).

The duration of symptoms also shortened with successive vaccinations, with the median decreasing from 3 days (IQR 2–5) after the first dose to just 1 day (IQR 1–2) after the fourth dose. Female participants consistently reported higher reactogenicity across all doses (OR 1.87; 95% CI 1.32–2.65; *p* < 0.001), whereas increasing age exhibited a weak negative correlation with the occurrence of adverse events (r = −0.23; *p* < 0.001).

### 3.3. Immunogenicity and Antibody Kinetics

Values represent cross-sectional medians of available participants at each timepoint. Median anti-SARS-CoV-2 IgG levels increased progressively with successive doses, rising from 9910 AU/mL (IQR 5234.2–18,645.3) after the primary series to 29,002 AU/mL (IQR 17,951.1–45,440.5) after the third dose, representing a 2.9-fold increase. Following the fourth dose, antibody concentrations reached 38,274 AU/mL (IQR 33,216.5–62,452.5), corresponding to a 3.9-fold increase relative to baseline.

Antibody half-life also extended with repeated vaccination, increasing from 91 days (95% CI 87–95) after the primary series to 126 days (95% CI 117–136) after the fourth dose (*p* < 0.001 for trend). Participants with hybrid immunity consistently demonstrated superior responses, maintaining 2.3-fold higher peak antibody levels compared with infection-naïve individuals (median 22,793 vs. 9910 AU/mL after the primary series; *p* < 0.001).

### 3.4. Correlation Between Reactogenicity and Immunogenicity

**Figure 2:**
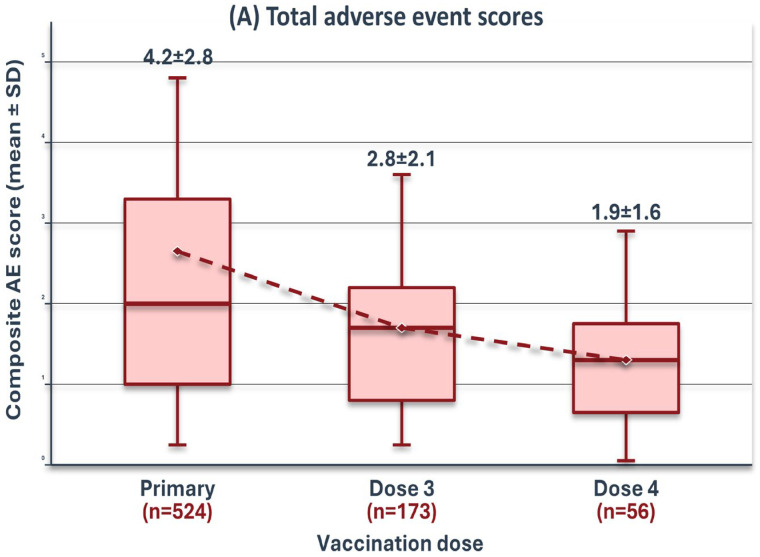

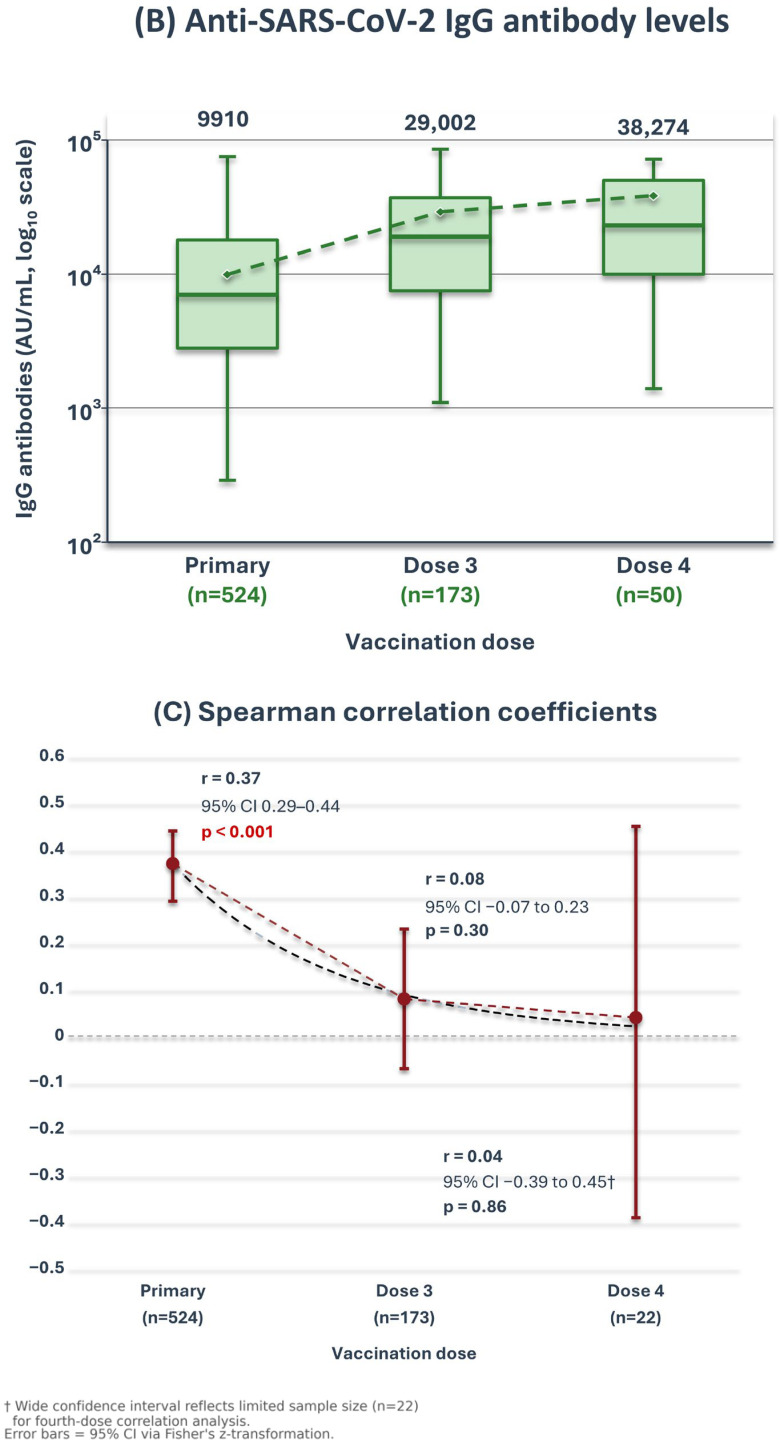
Progressive dissociation between reactogenicity and immunogenicity across four BNT162b2 vaccine doses. (**A**) Composite AE scores (mean ± SD) demonstrating progressive decrease from primary series through fourth dose (fourth dose: *n* = 56 participants). (**B**) Anti-SARS-CoV-2 IgG antibody levels on logarithmic scale showing progressive increase (fourth dose: *n* = 50 participants with antibody measurements). (**C**) Spearman correlation coefficients between composite AE scores and antibody titres, revealing a declining association (fourth dose: *n* = 22 participants with complete paired data).

The association between adverse events and antibody titers progressively weakened across vaccine doses (Table 2; Figure 2). After primary immunization, a moderate positive correlation was observed (r = 0.37; 95% CI 0.29–0.44; *p* < 0.001). This relationship declined substantially after the third dose (r = 0.08; 95% CI −0.07 to 0.23; *p* = 0.30) and approached zero following the fourth dose (r = 0.04; 95% CI −0.39 to 0.45; *p* = 0.86).

An exponential decay model (r = 0.370 × exp(−1.430 × (d−2))) provided a close descriptive fit to the data (R^2^ = 0.9934). The decay constant (λ = 1.430) indicated a 76% reduction in correlation with each successive dose. The model predicted r = 0.021 for the fourth dose, while the observed value was r = 0.04; both values indicate near-zero correlation consistent with an absence of detectable correlation within the limits imposed by the small sample size of the fourth-dose subgroup (*n* = 22), supporting the validity of the exponential decay pattern. Participants with hybrid immunity exhibited an earlier dissociation between reactogenicity and immunogenicity, already evident after the primary series (r = 0.12; 95% CI −0.05 to 0.28; *p* = 0.31).

### 3.5. Clinical Outcomes and Safety

No serious vaccine-related adverse events were documented during the 36-month follow-up period, underscoring the favorable long-term safety profile of repeated BNT162b2 administration. Breakthrough infections that occurred were uniformly mild and showed no correlation with antibody concentrations at the time of infection. No severe COVID-19 cases were observed among participants who received four doses.

### 3.6. Sensitivity Analyses

Several sensitivity analyses confirmed the robustness of our primary findings. Restriction to complete cases yielded correlation coefficients consistent with the primary analysis across all dose groups. Application of alternative correlation methods, including Kendall’s tau and Pearson correlation on log-transformed data, produced concordant results. When participants with breakthrough infections were retained in the analysis, the primary-series correlation remained significant and the progressive decline pattern was preserved. For the fourth-dose correlation, Bayesian estimation using weakly informative priors (Normal(0, 1)) yielded a posterior mean of 0.042, while skeptical priors (Normal(0, 0.05)) produced a posterior mean of 0.038, both consistent with the observed Spearman correlation (r = 0.04). Leave-one-out cross-validation of the exponential decay model demonstrated stable predictions (RMSE = 0.021). Little’s MCAR test suggested that data were missing completely at random (*p* = 0.423).

## 4. Discussion

This 36-month longitudinal study involving 524 Croatian healthcare workers not only documents but also demonstrates a progressive dissociation between reactogenicity and immunogenicity across four doses of the BNT162b2 vaccine. The correlation between adverse events and antibody response declined exponentially, from *r* = 0.37 (95% CI 0.29–0.44, *p* < 0.001) after the primary series to *r* = 0.04 (95% CI –0.39 to 0.45, *p* = 0.86) after the fourth dose, representing an 89% reduction. Concurrently, during this same period, antibody levels increased 3.9-fold while reactogenicity decreased by 55%, directly challenging the prevailing assumption that adverse events reliably predict immune response. The exponential decay model (*R*^2^ = 0.9934) provides a robust quantitative framework for this phenomenon and simultaneously opens new perspectives for booster strategies and vaccine communication.

Our findings are consistent with early reports of a positive correlation between reactogenicity and immunogenicity, yet they also reveal patterns that become apparent only with extended follow-up. Previous studies described comparable correlations after the primary vaccine series (e.g., *r* = 0.366), thereby confirming our baseline results [24]. Japanese investigations likewise identified associations between systemic reactions and antibody titers, although these were restricted to two-dose regimens [41,42]. Large-scale Israeli cohorts confirmed durable booster efficacy despite reduced reactogenicity, reporting a 90% reduction in COVID-19 mortality and 81–93% effectiveness against severe outcomes [43,44]. In contrast, several studies reported weak or absent associations: some detected no correlation [25,45], while others observed only marginal effects [46,47,48,49]. Large-scale pharmacovigilance data further indicate increased reactogenicity after initial doses [50], whereas adverse events following booster doses were generally of mild intensity [51,52]. Such discrepancies likely reflect methodological differences, population heterogeneity, and variation in assessment timing. Importantly, however, no previous investigation has systematically quantified correlation dynamics across four doses over a 36-month period, making our exponential decay model the first comprehensive contribution in this domain.

The progressive dissociation documented in our study may partly reflect the engagement of idiotypic network regulation, as described in the Jerne network hypothesis [53]. Anti-idiotypic antibodies (Ab2) serve as physiological regulators of the humoral response, modulating antibody production through feedback mechanisms rather than eliminating it [54]. The observation of maintained high antibody titers (median 38,274 AU/mL after dose 4) despite reduced reactogenicity is consistent with a maturing immune response where regulatory feedback achieves efficient antigen recognition without excessive inflammatory activation. Importantly, our study measured total anti-spike IgG binding capacity, which represents the net balance of idiotypic and anti-idiotypic regulation—the sustained high levels indicate that productive immunity is preserved despite regulatory modulation. Future studies incorporating anti-idiotypic antibody measurements alongside standard serological panels would provide valuable insight into this regulatory dimension of repeated vaccination.

The observed dissociation may be driven by several interconnected mechanisms, although our study design does not allow for direct mechanistic confirmation. At the molecular level, the N1-methylpseudouridine modification in BNT162b2 likely reduces inflammatory signaling while preserving antigen presentation [18]. At the humoral level, progressive IgG4 class switching, reported to rise from <1% to approximately 20% after repeated doses [21,22], could explain the maintenance of protection with minimal reactogenicity through reduced Fc-mediated inflammation. Immunosenescence may further contribute to age-related declines in reactogenicity, as diminished CD4+ T cell responses correlate with fewer systemic adverse effects in older adults [55]. The early dissociation observed in hybrid immunity participants (*r* = 0.30 vs. *r* = 0.37) suggests that pre-existing immunity may accelerate these adaptations, while immune imprinting from repeated antigen exposures could further shape B-cell responses [56]. Importantly, elevated IgG4 levels are simultaneously associated with an increased risk of breakthrough infections [23], highlighting the complex balance between protective immunity and susceptibility to infection. Beyond immunological mechanisms, sex-based differences provide additional insights into reactogenicity patterns. Females exhibited significantly higher reactogenicity across all doses (OR 1.87; 95% CI 1.32–2.65), consistent with sex-disaggregated analyses [57]. This heightened female response may be mediated by X-chromosome-linked TLR7 gene dosage effects, as TLR7 escapes X-inactivation, leading to increased type I interferon production [58]. These mechanisms remain hypothetical in the absence of direct immunophenotyping, but they underscore the multifactorial nature of vaccine responses.

The findings carry significant clinical implications for vaccination strategies. Clinicians can reassure patients that minimal side effects after booster doses do not indicate compromised protection. Our data demonstrate sustained high antibody levels (median 38,274 AU/mL) and an extended half-life (126 days) despite a 56% reduction in systemic reactions. This information is critical for addressing vaccine hesitancy, particularly among older adults who prioritize avoiding adverse events. Moreover, the documented reduction in reactogenicity may improve healthcare worker adherence to booster recommendations and reduce post-vaccination absenteeism. For personalized vaccination approaches, our model indicates that traditional reactogenicity-based immune assessment becomes unreliable after multiple doses. Instead, clinicians should rely on serological markers rather than symptom severity when evaluating vaccine responses in multi-boosted individuals. These insights are especially timely in light of recent regulatory developments. The FDA’s 2025 announcement mandating placebo-controlled trials for COVID-19 vaccines in healthy younger adults [27] underscores the need for a comprehensive understanding of vaccine responses across multiple doses. Our exponential decay model demonstrates that traditional reactogenicity-based endpoints may be insufficient when used in isolation for an accurate assessment of booster dose efficacy. Furthermore, our data support recent ACIP recommendations for additional doses in adults ≥65 years [28], showing that this vulnerable population can receive boosters with confidence in both their safety (reduced reactogenicity) and efficacy (sustained antibody responses).

Despite strengths including prospective design, 83% retention rate, and comprehensive 16-timepoint sampling over 36 months, several limitations warrant consideration. Reactogenicity data were collected through self-reported questionnaires, which are inherently subjective and may be influenced by recall bias and individual pain thresholds. Although daily reminders for seven consecutive days post-vaccination were employed to minimize recall bias (Section 2.6), and the composite scoring system provided standardized assessment, self-reported outcomes remain less precise than clinician-assessed endpoints. The small fourth-dose subgroup (*n* = 22 for correlation analysis) substantially limits statistical precision and may introduce selection bias, although Little’s MCAR test (*p* = 0.423) indicated that missing data were random and the strong descriptive fit of the exponential model (*R*^2^ = 0.9934) provides confidence in the observed trend. Measurement of total IgG without subclass analysis precluded direct confirmation of IgG4 class switching, highlighting the need for multi-omic approaches to identify biomarkers for personalized vaccination strategies. In addition, the healthcare worker population may limit generalizability to elderly or immunocompromised groups, underscoring the importance of comparative studies across vaccine platforms to determine whether progressive dissociation is specific to mRNA vaccines. The absence of cellular immunity assessment restricts a complete understanding of T-cell contributions to the observed dissociation pattern. While breakthrough infections occurred throughout follow-up (14.5%, 27.5%, and 5.4% after doses 2, 3, and 4, respectively), their limited number, particularly after dose 4 (*n* = 3), precluded formal statistical analysis of associations with antibody levels or reactogenicity profiles.

The development of autoimmune events after mRNA vaccination has been reported in the literature [59], but large population studies have not confirmed a significantly increased risk [60]. In this study, autoimmune events were not observed among the 1522 doses received (95% CI for event rate: 0–0.2%) and were not included in further analysis. Further extended follow-up after vaccination is needed for a comprehensive conclusion.

Collectively, these limitations emphasize that our 36-month observation period cannot capture ultra-long-term effects beyond the fourth dose and highlight the need for dedicated immunological surveillance studies with appropriate biomarker panels, as well as longitudinal studies correlating early reactogenicity profiles with clinical outcomes such as breakthrough infections and disease severity, and autoimmune phenomena.

## 5. Conclusions

This longitudinal study demonstrates a progressive dissociation between reactogenicity and immunogenicity across successive BNT162b2 vaccine doses. The inverse relationship, characterized by declining adverse events alongside increasing antibody responses, follows a predictable exponential pattern. This phenomenon may represent a reproducible pattern within this cohort, although its ultimate clinical significance will require confirmation through long-term outcome studies. Patients who experience minimal side effects after booster doses can be reassured regarding maintained antibody levels, while the possibility of altered immune response quality underscores the need for ongoing surveillance. Our mathematical model for characterizing dissociation progression provides a quantitative framework for understanding immunological adaptation to repeated mRNA vaccination. Taken together, these findings support the evidence-based development of sustainable long-term vaccination strategies and highlight the need to refine traditional correlates of protection in the era of advanced vaccine platforms.

## Figures and Tables

**Table 1:** Participant Characteristics and Reactogenicity Across Four BNT162b2 Vaccine Doses.

Characteristic	Dose 1 *n* = 524	Dose 2 *n* = 524	Dose 3 *n* = 173 *	Dose 4 *n* = 56 **	*p*-Value ***
**Demographic characteristics**					
**Age, years**	42 (35–51)	42 (35–51)	42 (36–52)	43 (37–50)	0.521
**Female**	428 (81.7%)	428 (81.7%)	137 (79.2%)	40 (71.4%)	0.642
**Previous COVID-19**	75 (14.3%)	75 (14.3%)	17 (9.8%)	10 (17.9%)	0.683
**Local reactions**					
**Any local reaction**	432 (82.4%)	325 (62.0%)	84 (48.6%)	24 (42.9%)	<0.001
**Pain**	425 (81.1%)	312 (59.5%)	81 (46.8%)	23 (41.1%)	<0.001
**Swelling**	63 (12.0%)	111 (21.2%)	31 (17.9%)	8 (14.3%)	0.004
**Erythema**	60 (11.5%)	94 (17.9%)	34 (19.7%)	6 (10.7%)	0.031
**Systemic reactions**					
**Any systemic reaction**	156 (29.8%)	235 (44.8%)	45 (26.0%)	11 (19.6%)	<0.001
**Fatigue**	69 (13.2%)	127 (24.2%)	25 (14.5%)	9 (16.1%)	<0.001
**Headache**	80 (15.3%)	129 (24.6%)	26 (15.0%)	7 (12.5%)	<0.001
**Myalgia**	87 (16.6%)	156 (29.8%)	22 (12.7%)	6 (10.7%)	<0.001
**Fever ≥38 °C**	21 (4.0%)	87 (16.6%)	22 (12.7%)	2 (3.6%)	<0.001
**Reaction severity**					
**Grade 3 (severe)**	30 (5.7%)	65 (12.4%)	8 (4.6%)	1 (1.8%)	<0.001
**Analgesic use**	142 (27.1%)	222 (42.4%)	68 (39.3%)	15 (26.8%)	<0.001
**Symptom duration, days**	3 (2–5)	2 (1–3)	2 (1–2)	1 (1–2)	<0.001

* Of 418 participants who received the third dose, 173 had complete adverse event and antibody level data for analysis. ** Of 56 participants who received the fourth dose, adverse event data shown are from all 56 participants. Note that correlation analysis (Table 2) used only *n* = 22 with complete paired data. *** *p*-values refer to trend across doses using the Cochran–Armitage test for categorical variables and the Jonckheere–Terpstra test for continuous variables. Data are *n* (%) for categorical variables or median (IQR) for continuous variables.

## Data Availability

Anonymized participant-level data supporting the results of this study will be available 3 months after publication upon reasonable request to researchers whose proposal is approved by an independent review board.

## Supplementary Materials

The following supporting information can be downloaded at: https://www.mdpi.com/article/10.3390/vaccines14040305/s1, Table S1. STROBE Statement—Checklist of items that should be included in reports of cohort studies.
