# Supplementary material for: Progressive Dissociation Between Reactogenicity and Immunogenicity After Four-Dose BNT162b2 Vaccination: A 36-Month Longitudinal Study
Source: Vaccines (Basel). 2026 Mar 28;14(4):305. doi: 10.3390/vaccines14040305 (PMC13119717; doi:10.3390/vaccines14040305)
Supplement: Supplementary file 1 [file vaccines-14-00305-s001.zip › vaccines-4181310-supplementary.pdf]

Supplementary Materials

Table S1. STROBE Statement—Checklist of items that should be included in reports of cohort studies

Item No.	Recommendation	Section	Relevant text from manuscript
**Title and abstract**			
1(a)	(a) Indicate the study's design with a commonly used term in the title or the abstract	Title	Title: "Progressive Dissociation Between Reactogenicity and Immunogenicity After Four-Dose BNT162b2 Vaccination: A 36-Month Longitudinal Study"
1(b)	(b) Provide in the abstract an informative and balanced summary of what was done and what was found	Abstract	Structured abstract (Background/Objectives/Methods/Results/Conclusions): prospective longitudinal cohort, n=631/524, Spearman r=0.37→0.04, exponential model R^2=0.9934, p-values and 95% CI throughout.
**Introduction**			
2	Explain the scientific background and rationale for the investigation being reported	Section 1	Background: mRNA vaccine mechanism, IgG4 class switching [21,22], prior correlation data (r=0.366, p<0.0001) [24], knowledge gap: "no study has systematically quantified this relationship across four consecutive doses in a multi-year follow-up."
3	State specific objectives, including any prespecified hypotheses	Section 1	"We hypothesize that repeated BNT162b2 vaccination induces a progressive dissociation." Primary: quantify correlation across 4 doses. Secondary: (1) antibody half-life, (2) hybrid immunity, (3) mathematical decay pattern.
**Methods**			
4	Present key elements of study design early in the paper	Section 2.1	We conducted a prospective longitudinal cohort study at the University Hospital for Infectious Diseases "Dr. Fran Mihaljević", Zagreb, Croatia, from 5 January 2021 to 31 January 2024. The study adhered to the Declaration of Helsinki [29] and was reported in accordance with STROBE guidelines [30] (Supplementary Materials, Table S1).
5	Describe the setting, locations, and relevant dates, including periods of recruitment, exposure, follow-up, and data collection	Section 2.1	Setting: UHID Zagreb. Recruitment: national COVID-19 campaign. Dates: 5 Jan 2021–31 Jan 2024. Follow-up: 16 predefined timepoints (3 wk post-dose 1 through 12 mo post-dose 4).

Item No.	Recommendation	Section	Relevant text from manuscript
6(a)	(a) Cohort study—Give the eligibility criteria, and the sources and methods of selection of participants. Describe methods of follow-up	Section 2.1	Inclusion: HCW aged >18 years, BNT162b2 recipients, consent to serial testing. Exclusion: non-BNT162b2, allergic reaction, pregnancy, incomplete data. Follow-up at 16 timepoints; daily reminders ×7 days.
6(b)	(b) Cohort study—For matched studies, give matching criteria and number of exposed and unexposed	N/A	Not applicable—not a matched study design.
7	Clearly define all outcomes, exposures, predictors, potential confounders, and effect modifiers. Give diagnostic criteria, if applicable	Sections 2.3–2.4	Primary outcome: composite AE score (FDA toxicity Grade 0–3). Immunogenicity: anti-SARS-CoV-2 IgG (AU/mL). Exposures: vaccine doses 1–4. Confounders: age, sex, prior infection. Diagnostics: WHO severity criteria [31].
8	For each variable of interest, give sources of data and details of methods of assessment (measurement). Describe comparability of assessment methods if there is more than one group	Sections 2.3–2.4	AE: standardized questionnaires + trained personnel + daily reminders. IgG: Abbott ARCHITECT SARS-CoV-2 IgG II Quant (CMIA), range 21–40,000 AU/mL, r=0.89 with neutralization [35]. Same assay across all timepoints.
9	Describe any efforts to address potential sources of bias	Section 2.6	Section 2.6: consecutive enrollment (selection bias), daily reminders ×7 days (recall bias), standardized questionnaires by trained personnel (information bias).
10	Explain how the study size was arrived at	Section 2.5	A priori power analysis: r≥0.30, 90% power, α=0.05, minimum n=112/group. Primary cohort n=524; third-dose n=173—both exceed the required minimum.
11	Explain how quantitative variables were handled in the analyses. If applicable, describe which groupings were chosen and why	Section 2.5	Categorical: n (%). Continuous: mean±SD or median (IQR) per Shapiro–Wilk. AE graded 0–3 (FDA toxicity scale). Antibodies as continuous AU/mL. Composite AE score = sum of individual grades.
12(a)	(a) Describe all statistical methods, including those used to control for confounding	Section 2.5	Spearman correlation + Fisher z-transformation for CI; Cochran–Armitage trend; Jonckheere–Terpstra; exponential model $r(d)=r_0\times e^{(-\lambda(d-2))}$; Bonferroni correction; OR with 95% CI. R v4.3.3 + tidyverse [39,40].

Item No.	Recommendation	Section	Relevant text from manuscript
12(b)	(b) Describe any methods used to examine subgroups and interactions	Section 2.5	Pre-specified subgroups: hybrid immunity vs. infection-naive; sex-stratified (OR 1.87, 95% CI: 1.32–2.65 for female sex); age correlation ($r=-0.23$, $p<0.001$).
12(c)	(c) Explain how missing data were addressed	Section 2.5	Little's MCAR test ($p=0.423$, confirmed MCAR). Double data entry + range checks. Sensitivity analysis: complete cases only.
12(d)	(d) Cohort study—If applicable, explain how loss to follow-up was addressed	Section 3.1; Figure 1	STROBE flow diagram (Figure 1): 631→524→418→56 with documented reasons at each stage. Breakthrough infection participants excluded from subsequent immunological analyses.
12(e)	(e) Describe any sensitivity analyses	Section 2.5	(1) Excluding participants with breakthrough infections; (2) complete cases only; (3) alternative correlation methods for robustness [38].
**Results**			
13(a)	(a) Report numbers of individuals at each stage of study	Section 3.1; Figure 1	631 enrolled → 107 excluded (9 allergic, 98 incomplete) → 524 primary → 418 dose 3 (173 complete pairs) → 56 dose 4 (22 complete pairs, 50 antibody, 56 AE).
13(b)	(b) Give reasons for non-participation at each stage	Section 3.1; Figure 1	9 allergic reactions (8 generalized rash with pruritus, 1 neurological symptoms); 98 incomplete data. All allergic participants recovered fully with antiallergic therapy.
13(c)	(c) Consider use of a flow diagram	Figure 1	STROBE flow diagram presented as Figure 1 with comprehensive legend describing participant progression through all study phases.
14(a)	(a) Give characteristics of study participants and information on exposures and potential confounders	Section 3.1; Table 1	Table 1: 81.7% female, median age 42 (IQR 35–51). AE rates, composite scores, antibody titers, and p-values across all 4 doses.
14(b)	(b) Indicate number of participants with missing data for each variable of interest	Section 3.1; Table footnotes	Fourth dose: 50/56 antibody data, 22/56 complete pairs, 56/56 AE. Little's MCAR $p=0.423$. Aggregate missing data in Results 3.1 and Table 2 footnotes.
14(c)	(c) Cohort study—Summarise follow-up time	Sections 2.1, 3.3	Total: 36 months (Jan 2021–Jan 2024). 16 predefined timepoints. Antibody half-life: 91 days (primary series) extending to 126 days (fourth dose) via nonlinear mixed-effects modeling [36].

Item No.	Recommendation	Section	Relevant text from manuscript
15	Cohort study—Report numbers of outcome events or summary measures over time	Sections 3.2–3.3; Tables 1–2	Table 1: local AE 82.4%→42.9%; systemic peak 44.8% (dose 2). Table 2: antibody 9910→29,002→38,274 AU/mL (2.9×/3.9× fold). Breakthrough: 76 (14.5%), 115 (27.5%), 3 (5.4%).
16(a)	(a) Give unadjusted estimates and, if applicable, confounder-adjusted estimates and their precision (eg, 95% confidence interval). Make clear which confounders were adjusted for and why they were included	Sections 3.2, 3.4; Table 2; Figure 2	Spearman r: dose 2 = 0.37 (95% CI: 0.29–0.44, $p<0.001$); dose 3 = 0.08 (−0.07 to 0.23, p=0.30); dose 4 = 0.04 (−0.39 to 0.45, p=0.86). Sex-adjusted OR 1.87 (95% CI: 1.32–2.65). Progressive dissociation across doses visualized in Figure 2.
16(b)	(b) Report category boundaries when continuous variables were categorized	Sections 2.1, 2.3	FDA toxicity: Grade 0 (none), 1 (mild), 2 (moderate), 3 (severe). Breakthrough: ≥14 days post-dose completion.
16(c)	(c) If relevant, consider translating estimates of relative risk into absolute risk for a meaningful time period	Section 3.1	Breakthrough infection rates as absolute percentages: 14.5%, 27.5%, 5.4% after doses 2, 3, 4. Primary outcome is correlation coefficient (Spearman r); formal relative-to-absolute risk translation not applicable.
17	Report other analyses done—eg analyses of subgroups and interactions, and sensitivity analyses	Sections 3.2, 3.4; Figure 2	Hybrid immunity: r=0.12 vs. naive r=0.37. Exponential model: R^2=0.9934, λ=1.430, predicted dose 4 r=0.021 (observed r=0.04). Sex: OR 1.87. Age: r=−0.23. Exponential decay model with observed vs. predicted correlation plotted in Figure 2.
**Discussion**			
18	Summarise key results with reference to study objectives	Section 4, para 1	Opening paragraph: r=0.37→0.04 across 4 doses (89% reduction); exponential model R^2=0.9934; 55% AE reduction; addresses all stated primary and secondary objectives.
19	Discuss limitations of the study, taking into account sources of potential bias or imprecision. Discuss both direction and magnitude of any potential bias	Section 4	Six limitations: (1) reduced n=22 for dose 4, (2) no IgG subclass analysis, (3) HCW population generalizability, (4) no cellular immunity, (5) limited breakthrough infections (n=3 post-dose 4), (6) 36-month observation period.
20	Give a cautious overall interpretation of results considering objectives, limitations, multiplicity of analyses, results from similar	Section 4	Cautious language: "may be driven by," "does not allow for direct mechanical confirmation," "remains hypothetical." Comparison with Levy et al. [24], Bauernfeind et al. [25], and 10+ additional references.

Item No.	Recommendation	Section	Relevant text from manuscript
	studies, and other relevant evidence		
21	Discuss the generalisability (external validity) of the study results	Section 4	"Studying a healthcare worker population may limit generalizability to older or immunocompromised groups, underscoring the importance of comparative studies across vaccine platforms."
**Other information**			
22	Give the source of funding and the role of the funders for the present study and, if applicable, for the original study on which the present article is based	Funding	Grant "Strengthening the capacity of CerVirVac" (KK.01.1.1.01.0006), co-financed by the Croatian Government and the European Regional Development Fund.

Declaration
We confirm that this manuscript has been prepared in accordance with the STROBE (Strengthening the Reporting of Observational Studies in Epidemiology) guidelines for cohort studies. The checklist demonstrates adherence to established reporting standards for observational research.

STROBE Statement Reference: von Elm E, Altman DG, Egger M, Pocock SJ, Gøtzsche PC, Vandenbroucke JP; STROBE Initiative. The Strengthening the Reporting of Observational Studies in Epidemiology (STROBE) statement: guidelines for reporting observational studies. Lancet. 2007;370(9596):1453–1457. https://doi.org/10.1016/S0140-6736(07)61602-X
